# Metformin Renders Survival Advantage to Patients with Glioblastoma Multiforme

**DOI:** 10.3390/neurolint18030040

**Published:** 2026-02-24

**Authors:** Daniel Gonzales-Portillo, Bhavya Vashi, Kirsten Bains Williams, Jorge Cervantes

**Affiliations:** 1 Kiran C. Patel College of Allopathic Medicine, Nova Southeastern University, Fort Lauderdale, FL 33328, USA; 2 Florida Atlantic University, Boca Raton, FL 33328, USA

**Keywords:** metformin, glioblastoma multiforme, cancer therapy

## Abstract

**Purpose**: Glioblastoma multiforme (GBM) is a highly aggressive cancer with limited survival despite current treatments. Rising treatment costs highlight the importance of identifying more affordable therapeutic alternatives. A body of literature has shown that metformin has the potential to act as an antineoplastic agent. Here, we examined the effects of metformin on GBM in humans. **Methods**: The Preferred Reporting Items for Systematic reviews and Meta-Analyses (PRISMA) guidelines were followed to perform the review. A total of 469 studies were screened using comprehensive search terms. Of these, 4 studies were compatible for the meta-analysis. **Results**: Data analysis demonstrated an increase in median overall survival for GBM patients up to 18 months compared to controls (*p* = 0.00197). **Conclusions**: Overall, our findings support the efficacy of metformin as an anti-neoplastic agent, and that it may grant a survival advantage for patients diagnosed with GBM. Further analyses should find dose-dependent relationships between metformin and the targeted survival outcomes in larger, rigorous clinical trials.

## 1. Introduction

Among primary malignant adult brain tumors, glioblastoma multiforme (GBM) is both the most common and aggressive, accounting for roughly half of all malignant central nervous system tumors [1]. Although GBM arises most commonly in brain parenchyma, it can also develop in the brainstem, cerebellum, and spinal cord. Treatment strategies are multimodal, including a mixture of surgical resection, radiation, and chemotherapy. However, outcomes remain dismal with frequent recurrence. This treatment paradigm is associated with a median survival of 14 to 16 months after diagnosis, and long-term survival beyond 5 years is uncommon [2]. Because of this poor outlook, there is a strong need to find new and better treatment options.

Modern cancer treatments, particularly targeted therapies and immunotherapies have shown promise. However, costs have continued to rise excessively [3]. Such challenges have intensified efforts to explore the potential of repurposing inexpensive, commonly used drugs as anticancer agents. One of these drugs is metformin, which is widely used to treat type 2 diabetes and has a well-known safety profile [4].

In recent years, metformin’s effects have been found to extend beyond glycemic control, with novel antineoplastic and immunomodulatory properties coming to light. Previous studies have demonstrated how metformin can interrupt cellular metabolism and growth signaling through its actions on mitochondrial respiration and the mTOR signaling pathway [5]. This disruption can slow cancer growth by halting proliferation and survival pathways [6]. Since GBM is a highly active and treatment-resistant tumor, these effects may be especially beneficial.

The effect of metformin on GBM has been successful in animal models. Some studies have examined metformin alone as an antineoplastic agent, where metformin-treated mice had a median overall survival of 22.71 days, compared to 20 days in the no treatment group [7]. When used as an adjuvant therapy in murine models, metformin extended the median survival of mice to 23 days compared to 18 days for Temozolomide (TMZ) alone in mice with TMZ resistant GBM [8]. It has also shown to enhance PD-1 immunotherapies, significantly increasing median overall survival compared to control or PD-1 therapy alone [9].

A few observational studies in humans suggest that people with brain tumors who also take metformin might live longer than those who do not [10,11]. However, the results from these studies remain inconsistent. We here conducted a systematic review and meta-analysis to better understand whether metformin helps improve median overall survival in people with GBM.

## 2. Material and Methods

### 2.1. Protocol and Registration

This meta-analysis followed the PRISMA (Preferred Reporting Items for Systematic Reviews and Meta-Analyses) guidelines (Table S1). Registered in the International Prospective Register of Systematic Reviews, PROSPERO (Registration ID: CRD42024575930).

### 2.2. Search Strategy and Information Sources

Several databases, including PubMed, EMBASE, and MEDLINE with Full Text using the EBSCOhost platform, were used for the literature search. In addition, a search in CINAHL Complete, Biomedical Reference Collection: Comprehensive, and Cochrane Clinical Answers was performed. The search included all studies available up to January 2025. There were no limits on publication dates or language. We included randomized controlled trials (RCTs), as well as prospective or retrospective observational studies. There were no limits on publication dates or languages, and duplicates were removed.

Studies that involved human patients diagnosed with GBM, regardless of age, sex, or diabetic status were included. Studies were eligible if they looked at metformin either as a main treatment or as an added treatment along with standard chemotherapy. Patients receiving standard therapy without metformin constituted the control group. The main outcome of interest was the median overall survival.

Our search string for human trials on PubMed was ((Metformin) AND (Glioblastoma Multiforme)) AND (human). Our search string on EMBASE was ‘glioblastoma’/exp AND ‘metformin’/exp AND ‘human’/exp. The search string across all other databases using EBSCOhost was (metformin) AND (glioblastoma multiforme or gbm) AND (human). All duplicates were removed from the pulled studies, and reviews were filtered out. Each study was screened through their title and abstracts, and a second round of review was performed using the full text.

### 2.3. Data Extraction and Statistical Analysis

Two reviewers independently screened all titles and abstracts to decide which studies were relevant. After that, full-text versions of potentially eligible studies were reviewed. For each study, information was recorded on design, sample size, treatment regimen, metformin dosage, median overall survival, and hazard ratios.

Hazard ratios (HRs) were used to assess the effect of metformin on median overall survival. We utilized the data from the studies to generate a Kaplan–Meier curve. A Cox proportional hazards model was used to estimate the effect of metformin on survival.

Statistical analysis of pooled survival was conducted in R software (Version 4.4.1), and a random-effects model was utilized to account for heterogeneity across studies.

## 3. Results

**Figure 1:**
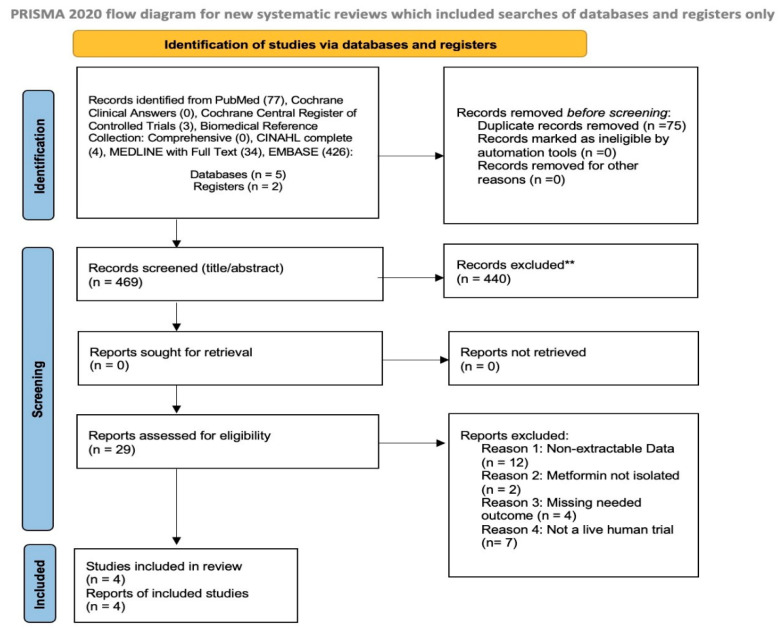
PRISMA Flow diagram for human trials. ** If automation tools were used, indicate how many records were excluded by a human and how many were excluded by automation tools. It is what the Prisma output showed.

Our search yielded 4 trials that were compatible with our analysis. These studies ranged from 2020 to 2023. A PRISMA flow diagram illustrates the process of study selection (Figure 1). A total of 541 studies were pulled on the initial search, and 75 duplicates were removed. After screening through the articles by abstract and title, 29 articles remained for full-text review. In the end, 4 articles were eligible for the meta-analysis.

Table 1 shows the type of study, which mutations were included or excluded, and the overall result from each study. Three of the four studies included patients with MGMT methylation, while only one included a known IDH mutation. Three of the studies are retrospective cohort studies, and one reported data from a randomized control trial.

**Figure 2:**
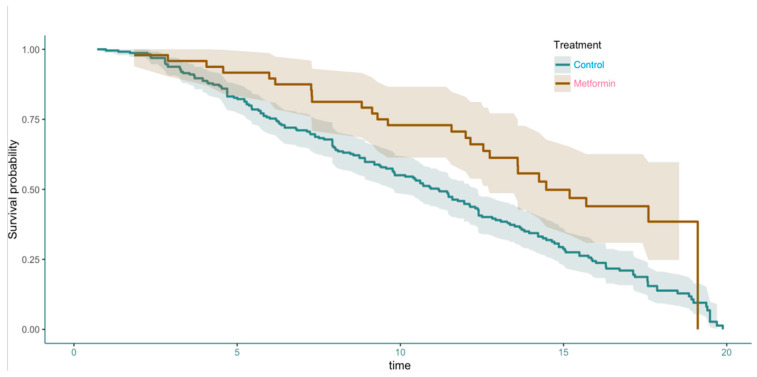
Pooled data survival analysis showed an increased survival in GBM patients taking Metformin (Brown line) compared to controls (Green line). *p* < 0.00197 (Cox Proportional Hazards Test).

We first analyzed the effect of metformin on the overall survival of GBM patients. Pooled data survival analysis showed an increased survival in GBM patients taking metformin compared to controls (*p* < 0.00197, Cox Proportional Hazards Test) (Figure 2). The effect was most notable between 8 and 18 months. At 18 months a dramatic drop in the survival of patients receiving metformin was observed.

**Figure 3:**
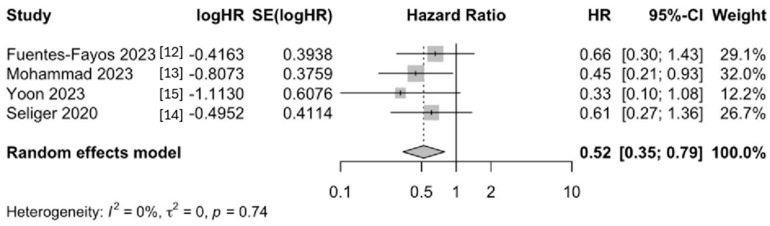
Forest plot of the hazard ratios for overall survival comparing GBM patients treated with metformin to controls.

We then analyzed the effect size on survival outcomes and found that survival was better in GBM patients who received metformin across multiple studies. The random-effects model demonstrated a significant improvement in survival among the metformin-treated groups (HR [CI], 0.52 [0.35, 0.79]) (Figure 3).

## 4. Discussion

Our pooled analysis of the effect of metformin across human trials showed a statistically significant increase in median overall survival. Despite the observed improvements in median survival, this effect was not sustained over an extended period of time. It is possible that other prognostic factors, such as isocitrate dehydrogenase (IDH) mutation status or O^6^-methylguanine-DNA methyltransferase (MGMT) methylation [16,17], could be affecting metformin’s effect. Further investigation is needed to identify the ideal conditions in which Metformin can effectively contribute to GBM patients’ survival.

Metformin is a very inexpensive drug that could enhance median overall survival while adding minimal financial burden. The mean cost of modern GBM treatment, including craniotomy, radiation, and chemotherapy, is relatively expensive while achieving a median overall survival of around 16 months [18]. Enhancing cost-effective care is essential in the modern healthcare landscape, where resources are finite and expensive. Metformin could be used as an adjuvant therapy in this setting.

We intended to perform a sub-group analysis comparing the efficacy of Temozolomide, a common chemotherapeutic drug used in GBM, to metformin. However, only one study reported data that included Temozolomide as its own group. We also observed a lack of standardized therapy across studies. In addition, each clinical trial participant had their best plan of care, with additional methods according to their randomization, for ethical reasons. As a result, each study’s baseline was a different mix of surgery, chemotherapy, or radiation at baseline. This limited the number of studies that met the inclusion criteria and analysis.

The tumor’s microenvironment plays an active role in driving GBM progression, therapy resistance, and immune evasion. Tumor-associated macrophages and microglia are polarized to secrete growth factors, cytokines, and chemokines [19] that ultimately promote tumor invasion, angiogenesis, and immune escape [20]. Metformin appears to have an effect on the viability of GBM cells via induction of cell death, as well as an immunomodulatory effect [19]. A decrease in mediators that contribute to growth and immunosuppression is observed upon MTF treatment of GBM cells [19]. Metformin is also able to block key signaling pathways involved in stemness transformation and invasiveness [21].

Our analysis reinforces prior evidence of metformin’s antineoplastic properties and suggests a potential survival benefit for patients with GBM. Metformin’s established safety and low cost make it a promising candidate to join current GBM treatment regimens. Establishing more rigorous clinical trials will help to establish its true role in anticancer therapy.

## Figures and Tables

**Table 1:** Characteristics of Included Studies.

Source	Type of Study	Included Mutation Status	Results
Fuentes-Fayos 2023 [12]	Retrospective Cohort	* IDH1	Metformin increased overall median survival compared to patients who did not receive it.
Mohammad 2023 [13]	Retrospective Cohort	** MGMT Excluded IDH1	Metformin was associated with increased survival for patients, particularly for those with known MGMT promotor methylation.
Seliger 2020 [14]	Retrospective Cohort	MGMT	Metformin was not associated with an increase in overall survival.
Yoon 2023 [15]	Randomized Control	MGMT	Metformin was not associated with an increase in overall survival.

*: Isocitrate Dehydrogenase 1; **: O6-methylguanine-DNA-methyltransferase.

## Data Availability

Data available on request from the authors.

## Supplementary Materials

The following supporting information can be downloaded at https://www.mdpi.com/article/10.3390/neurolint18030040/s1. Table S1: PRISMA 2020 Checklist [22].
